# Nonenzymatic and Trophic Activities of Carboxypeptidase E Regulate Bone Mass and Bioenergetics of Skeletal Stem Cells in Mice

**DOI:** 10.1002/jbm4.10392

**Published:** 2020-08-12

**Authors:** Amit Chougule, Vipula Kolli, Sudipta Baroi, Nabil Ebraheim, Piotr J Czernik, Y Peng Loh, Beata Lecka‐Czernik

**Affiliations:** ^1^ Department of Orthopaedic Surgery University of Toledo, College of Medicine and Life Sciences Toledo OH USA; ^2^ Department of Physiology and Pharmacology University of Toledo, College of Medicine and Life Sciences Toledo OH USA; ^3^ Center for Diabetes and Endocrine Research University of Toledo, College of Medicine and Life Sciences Toledo OH USA; ^4^ Section on Cellular Neurobiology Eunice Kennedy Shriver National Institute of Child Health and Human Development, National Institutes of Health Bethesda MD USA

**Keywords:** BIOENERGETICS, BONE MASS, CARBOXYPEPTIDASE E, INTRACELLULAR LIPIDS, OSTEOGENESIS

## Abstract

Bone and energy metabolism are integrated by common regulatory mechanisms. Carboxypeptidase E (CPE), also known as obesity susceptibility protein or neurotrophic factor‐α1, is recognized for its function in processing prohormones, including proinsulin and pro‐opiomelanocortin polypeptide. Independent of its enzymatic activity, CPE may also act as a secreted factor with divergent roles in neuroprotection and cancer growth; however, its role in the regulation of bone mass and skeletal cell differentiation is unknown. Male mice with global deficiency in CPE are characterized with profound visceral obesity, low bone mass in both appendicular and axial skeleton, and high volume of marrow fat. Interestingly, although metabolic deficit of CPE KO mice develops early in life, bone deficit develops in older age, suggesting that CPE bone‐specific activities differ from its enzymatic activities. Indeed, mutated CPE knockin (mCPE KI) mice ectopically expressing CPE‐E342Q, a mutated protein lacking enzymatic activity, develop the same obese phenotype and accumulate the same volume of marrow fat as CPE KO mice, but their bone mass is normal. In addition, differentiation of marrow hematopoietic cells toward tartrate‐resistant acid phosphatase‐positive multinucleated osteoclasts is highly increased in CPE KO mice, but normal in mCPE KI mice. Moreover, in murine skeletal stem cells, nonenzymatic trophic CPE has activated ERK signaling, increased cell proliferation and increased mitochondrial activity. Treatment of preosteoblastic cells with intact or mutated recombinant CPE led to a transient accumulation of small lipid droplets, increased oxidative phosphorylation, and increased cellular dependence on fatty acids as fuel for energy production. In human marrow aspirates, CPE expression increases up to 30‐fold in osteogenic conditions. These findings suggest that nonenzymatic and trophic activities of CPE regulate bone mass, whereas marrow adiposity is controlled by CPE enzymatic activity. Thus, CPE can be positioned as a factor regulating simultaneously bone and energy metabolism through a combination of shared and distinct mechanisms. © 2020 The Authors. *JBMR Plus* published by Wiley Periodicals, Inc. on behalf of American Society for Bone and Mineral Research © 2020 The Authors. *JBMR Plus* published by Wiley Periodicals LLC. on behalf of American Society for Bone and Mineral Research.

## Introduction

Bone and energy metabolism share many mechanisms operating on different levels, including cellular metabolism, cell differentiation, and function. Recent progress in our understanding of bone remodeling from a perspective of energy requirements of bone‐forming osteoblasts and bone‐resorbing osteoclasts has uncovered new potential targets to treat simultaneously pathological conditions associated with impairments in energy metabolism and bone metabolism. Carboxypeptidase E (CPE) is one of these targets.

CPE or neurotrophic factor‐α1 was discovered in 1982 as a prohormone‐processing enzyme with exopeptidase activity cleaving C‐terminal basic amino acid residues from prohormone intermediates to produce mature peptide hormones and neuropeptides in the endocrine and central nervous systems.^(^ ^1^, ^2^ ^)^ CPE is localized primarily to endocrine tissues and to specific areas of the central nervous system. As such, CPE is involved in processing proinsulin to insulin in pancreas and pro‐opiomelanocortin (POMC) to alpha melanocyte stimulating hormone (αMSH) and β‐endorphins in hypothalamus.^(^ ^3^, ^4^ ^)^

The importance of CPE as a processing enzyme was first realized when mutation in the *Cpe* gene was found in CPE^*fat/fat*^ mice, which were characterized by severe obesity, diabetes, and infertility.^(^ ^4^ ^)^ In addition to metabolic deficit observed in the CPE^*fat/fat*^ model, mice with total deficiency in CPE protein caused by deletion of exon 3 and 4 from a *Cpe* gene coding sequence developed depressive‐like behavior, impaired cognitive function, and low bone mass.^(^ ^5^, ^6^, ^7^ ^)^ Similarly, a null mutation in the *Cpe* gene locus in a human is associated with obesity, infertility, and learning deficits.^(^ ^8^ ^)^ There is no information, however, on the bone status of patients carrying mutations in *Cpe* gene locus.

Besides activities localized to peptidase enzymatic domain, CPE protein possesses other domains, including the prohormone binding and trafficking domain and the C‐terminal membrane binding domain. These domains determine the endogenous function of CPE, which consists of the sorting, transporting, and processing of neuropeptides and prohormones.^(^ ^5^ ^)^ Recently, CPE has been shown to act as an extracellular signaling molecule independently of its enzymatic activity. Secreted CPE protein promotes neurons cell survival during oxidative stress,^(^ ^9^, ^10^ ^)^ and inhibits proliferation of embryonic cortical stem cells while promoting their differentiation to astrocytes.^(^ ^11^ ^)^ Hence, it was given the alternate name of neurotrophic factor‐α1. Mechanistically, in neurons or neuronal stem cells, the extracellular actions of CPE activate the ERK signaling pathway, leading to either stimulation of *Bcl2* expression for neuroprotection^(^ ^9^ ^)^ or increased phosphorylation of SOX9 to mediate differentiation to astrocytes.^(^ ^11^ ^)^ It is postulated that CPE inhibits neuronal embryonic cortical stem cell proliferation by the mechanism involving the negative regulation of the WNT pathway and β‐catenin activity in these cells.^(^ ^11^ ^)^

A role of CPE in bone remodeling has been suggested before; however, it was more by association than activity. It has been shown that *Cpe* expression is relatively high in bone, which is consistent with the high expression in rat developing skeletal structures^(^ ^12^ ^)^ and rat growth plate chondrocytes from the perichondral zone and reserve zone.^(^ ^13^ ^)^ Moreover, it has been shown that CPE expression is upregulated during osteoclast differentiation induced by RANKL.^(^ ^14^ ^)^ However, CPE function in marrow cells of mesenchymal lineage or skeletal stem cells (SSCs) has not been studied.

In this study, we have revisited a role of CPE in the regulation of bone homeostasis. With the recent discoveries of the enzyme‐independent CPE activities regulating cell proliferation and differentiation combined with, as presented here, identification of *Cpe* transcript as a positive biomarker of human SSC differentiation to osteoblasts and a negative biomarker of murine SSC differentiation to adipocytes, we have asked the following questions: (i) whether CPE metabolic and skeletal activities are separate; (ii) what is the contribution of CPE enzymatic and nonenzymatic activities to the regulation of bone mass; (iii) whether CPE plays a direct role in the regulation of osteoclast, osteoblast, and adipocyte differentiation; and (iv) whether CPE regulates osteoblast bioenergetics.

## Materials and Methods

### Animals

CPE KO, mutated CPE knockin (mCPE KI) and control WT mice were developed at the NIH/National Institute of Child Health and Human Development under the IACUC protocol, #17–024, and transferred to the University of Toledo (Toledo, OH, USA). Animal experiments carried at the University of Toledo were performed using the University of Toledo Health Science Campus IACUC protocol. All animals were on the C57BL/6 background. The CPE KO animals have deleted exons 3 and 4 of the *Cpe* gene, which results in a total absence of CPE protein,^(^ ^15^ ^)^ whereas mCPE KI mice are CPE KO mice carrying a construct coding for CPE protein lacking enzymatic activity caused by a point mutation substituting glutamic acid with glutamine at the position 342 (E342Q). The E342Q mutation obliterates CPE enzymatic activity, leaving nonenzymatic domains of the protein intact. Both CPE KO and mCPE KI strains are infertile; homozygous offspring resulting from breeding heterozygotic parents are born with lower than expected frequency. The colonies of CPE KO, mCPE KI, and WT mice were housed with a 12‐hour dark–light cycle, and had free access to water and standard chow (PicoLab Mouse Diet 20, #5058; LabDiet, Fort Worth, TX, USA). All experiments were performed on 8‐ or 40‐week‐old male mice.

### Bone analysis

μCT of the tibias was performed using the μCT‐35 system (Scanco Medical AG, Bassersdorf, Switzerland), as previously described.^(^ ^16^ ^)^ Briefly, scans were performed at 70‐peak kilovoltage (kVp) energy and 113‐μA intensity settings and using 7‐μm voxel. Images of trabecular bone were segmented at a 289 threshold value using the per mille scale. The analysis of bone microstructure conformed to recommended guidelines.^(^ ^17^ ^)^

For lipid evaluation, decalcified bone specimens were stained for 1 hour in solution containing 2% osmium tetroxide prepared in 0.1M sodium cacodylate buffer, pH 7.4, according to the protocol.^(^ ^16^ ^)^ Staining was carried out in an exhaust hood and away from light because of osmium tetroxide toxicity and light sensitivity. Images of lipid depositions were acquired at 70‐kVP and 113‐μA settings and 12‐μm nominal resolution. Image segmentation was done under global threshold conditions by applying a gray‐scale threshold of 480 to 1000 using the per mille scale with the 3D noise filter set to sigma 1.2 and support 2.0. Lipid volumes were calculated directly from individual voxel volumes in 3D reconstructions.

### Human marrow specimen collection and cultivation of skeletal stem cells

The specimens were collected from human donors undergoing surgical procedures at the University of Toledo Medical Center. This study was approved by the University of Toledo Institutional Review Board protocol IRB #106541. Specimens were collected either in the form of discarded tissues or iliac crest aspirates, as described in Table 1. Isolation of adherent to plastic cells and their expansion were carried out using published methods with minor modifications.^(^ ^18^, ^19^ ^)^ Briefly, all cultures were maintained in α‐MEM supplemented with 15% FBS and 1% penicillin/streptomycin solution, and incubated at 37°C in 5% CO_2_ humidified atmosphere. Homogenized bone marrow aspirates were plated at cell density of 50,000 cells/cm^2^ excluding erythrocytes, which were removed by Zapoglobin during the initial cell counting. Bone tissues from femoral head or knee were cut into small pieces and immediately placed in the medium. Typically, dividing cells were becoming noticeable under microscope within 3 to 6 days and populating a 30‐mm culture dish to approximately 60% confluence in about 14 days. Next, cells were expanded in two to three passages and immediately used for experimentation. Cells were seeded at the density of 500 cells per cm^2^, in osteogenic media (α‐MEM supplemented with 10% FBS and 1% of penicillin/streptomycin solution, and 10mM β‐glycerophosphate, 50 μg/mL L‐ascorbic acid and 10nM dexamethasone) in either 96‐well plates for measuring alkaline phosphatase (ALP) activity or 60‐mm plates for RNA isolation. Identical parallel cultures were set‐up for cell proliferation assay [3‐(4,5‐dimethylthiazol‐2yl)‐2,5‐diphenyltetrazolium bromide] to ensure that a compensation for different cell growth rates is taken into account when comparing ALP activities among all SSC cultures. ALP and 3‐(4,5‐dimethylthiazol‐2yl)‐2,5‐diphenyltetrazolium bromide cell proliferation assays were conducted as previously described^(^ ^20^ ^)^ after 1, 3, 6, 9, and 12 days of cell growth. RNA was isolated from day 12 to correlate with ALP measurements after the same period.

**Table 1:** List of Human Specimens and Skeletal Stem Cell Isolates

Cell line	Donor's age	Sex	Harvest site	Source of cells
YB	93	F	Femoral head	Yellow (fatty) marrow
CT	72	M	Knee	Cartilage
MR	72	M	Knee	Red (hematopoietic) marrow
MA3	59	F	Iliac crest	Marrow aspirate
MA4	58	M	Iliac crest	Marrow aspirate

### Differentiation assays of murine bone marrow stromal cells

#### *Osteoclastogenesis*

Bone marrow nucleated cells were seeded at a density of 2.5 × 10^5^/cm^2^ in the presence of α‐MEM (Invitrogen, Carlsbad, CA, USA) supplemented with 15% FBS (Hyclone, Waltham, MA, USA). Floating, nonadherent cells were harvested after 24 hours and seeded at the density of 2 × 10^5^/cm^2^ in a 48‐well plate with the medium supplemented with macrophage colony‐stimulating factor (M‐CSF; 50 ng/mL) and RANKL (50 ng/mL; R&D System, Minneapolis, MN, USA). After 6 days of growth, cultures were stained for tartrate resistant acid phosphatase (TRAP) using the Leukocyte Acid Phosphatase (TRAP+) kit (Sigma Aldrich, St. Louis, MO, USA). Cells positive for TRAP staining with four or more nuclei were counted as osteoclasts. Counting was done in quadruplets.

#### *CFU‐AD and CFU‐OB assays*

Bone marrow nucleated cells were seeded at a density of 2.5 × 10^6^ cells/well in 6‐well plates in triplicates for each animal (*n* = 4 per group). For adipocyte differentiation, adherent bone marrow stromal cells (BMSCs) were grown for 10 days followed by 3 days in media supplemented with 1μM rosiglitazone (Sigma‐Aldrich, St. Louis, MO, USA). At day 13, cultures were stained for fat with oil red O and counterstained with methyl green, as described previously.^(^ ^21^ ^)^ Colony‐forming units for adipocytes (CFU‐ADs) were enumerated, considering CFU‐ADs a colony that contained at least 10% oil red O‐positive cells. For osteoblast differentiation, BMSCs were plated as above in media supplemented with 0.2mM ascorbic acid and 10mM β‐glycerophosphate, and maintained for 28 days with half the medium changed every 6 days. Colony‐forming units for osteoblasts (CFU‐OBs) were detected by Von Kossa staining of mineral, as described.^(^ ^21^ ^)^

#### *RNA for gene expression analysis*

BMSCs were grown in six‐well plates as above. RNA was isolated using TRIzol (Molecular Research Center Inc., Cincinnati, OH, USA) after 10 days of growth in basal media.

### Cell culture experiments

Osteoblastic cell lines U33^(^ ^22^ ^)^ and MC3T3 were grown on α‐MEM (Life Technologies Corp., Grand Island, NY, USA) supplemented with 10% FBS (HyClone Laboratories Inc., Utah, USA). Treatment with CPE proteins, intact rCPE, or mutated rCPE (E342Q [mrCPE]) proteins, were custom produced by Genscript (Piscataway, NJ, USA). The proteins were more than 95% pure. U33 or MC3T3 cells were seeded at a density of 0.5 × 10^4^/cm^2^ and treated with 100 ng/mL of either rCPE or mrCPE. After 24 hours, cells were stained for lipids with either oil red O or Nile red. For oil red O, cells were fixed with 10% formalin for 10 min, followed by staining for 1 hour, and brightfield microscopic images were taken. For Nile red, cells were fixed with 10% formalin for 10 min followed by 5‐hour staining with 0.05 μg/mL Nile red in the dark and images were taken by Cytation 5 (BioTek Instruments, Winooski, VT, USA) at excitation/emission wavelength of 549/575 nm. For gene‐expression analysis, cells were plated at a density 3 × 10^4^/cm^2^ in 6‐well plates and after 24‐hour treatment with CPE proteins, RNA was isolated and analyzed using qRT‐PCR. For analysis of pERK/ERK levels, cells were plated at the same density on 60‐mm plates, serum starved for 3 hours, and treated with 100 ng/mL rCPE or mrCPE from 10 to 25 min, as indicated, following protein isolation using CelLytic extraction reagent (Sigma‐Aldrich) supplemented with phosphatase inhibitors (PhosSTOP; Roche, Mannheim, Germany).

### Gene expression analysis using qRT‐PCR analysis

One μg of total RNA was converted to cDNA using the Verso cDNA Synthesis kit (Thermo Fisher Scientific, Waltham, MA, USA). PCR amplification of the cDNA was performed by qRT‐PCR using TrueAmp SYBR Green qPCR SuperMix (Smart Bioscience, Maumee, OH, USA) and processed with StepOne Plus System (Applied Biosystems, Carlsbad, CA, USA). The thermocycling protocol consisted of a single 10‐min step at 95°C, 40 cycles of 15 s at 95°C and 60 s at 60°C primer‐specific temperature, followed by a melting curve stage ranging from 60°C to 95°C to allow for evaluation of the product specificity. Relative gene expression was measured by the comparative ΔΔCT method using *18S* RNA levels for normalization. Primers were designed using OligoPerfect Designer (Thermo Fisher Scientific) and are listed in Supplementary Table S1.

### Gel electrophoresis and Western blotting

Protein samples were resolved by SDS‐polyacrylamide gel electrophoresis and electrophoretically transferred to immobilon‐FL membranes. Membranes were blocked at room temperature for 1 hour in TBS (10 mm Tris–HCl, pH 7.4, 150 mm NaCl) containing 3% BSA plus phosphatase inhibitors. Incubation with primary antibody was done overnight at 4°C. After three washes in TBST (TBS plus 0.1% Tween 20), membranes were incubated with either infrared anti‐rabbit (IRDye 800; green) or anti‐mouse (IRDye 680; red) secondary antibodies (LI‐COR Biosciences, Lincoln, NE, USA) at 1:10,000 dilution in TBS for 2 hours at 4°C. Immunoreactivity was visualized and quantified by infrared scanning in the Odyssey system (LI‐COR Biosciences). Polyclonal antibodies against total ERK1/2 (cat#9102) and monoclonal pERK1/2 antibodies (cat#9106) were obtained from Cell Signaling Technology (Beverly, MA, USA).

### Mitochondrial membrane potential

Active mitochondria were measured with TMRE Mitochondrial Membrane Potential Assay kit (Abcam, Cambridge, UK). U33 cells were seeded in 24‐well plates at a density of 25,000 cells/well and allowed to grow for 24 hours. Cells were treated with 100 ng/mL of either rCPE or mrCPE protein for 24 hours, followed by a change of media supplemented with 40nM TMRE (tetramethylrhodamine ethyl ester perchlorate). Cells were incubated for 20 min, media were aspirated, and replaced with 100‐mL PBS/0.2% BSA (twice), according to the manufacturer's protocol. The fluorescent intensity of TMRE accumulated in active mitochondria was measured using the Cytation 5 plate reader (BioTek Instruments) at excitation/emission wavelengths of 549/575 nm.

### Mito stress and fuel flex assays

For both the Seahorse XF Cell MitoStress test and Mito FuelFlex test (Seahorse Bioscience, Billerica, MA, USA), U33 cells were plated on the XF96 plates at the density of 10,000 cells/well and incubated overnight at 37°C. The next day, cells were treated with 100 ng/mL of either rCPE or mrCPE protein for 24 hours, followed by a medium change to XF assay medium loaded with 25mM glucose, 1mM pyruvate, and 2mM glutamine. For the FuelFlex test, XF media were additionally supplemented with either UK5099 to inhibit glucose oxidation, or etomoxir to inhibit fatty acid oxidation, or N,N'‐[thiobis(2,1‐ethanediyl‐1,3,4‐thiadiazole‐5,2‐diyl)]bis‐benzeneacetamide BPTES to inhibit glutamine oxidation, according to the manufacturer's protocol. The MitoStress and FuelFlex tests were performed using the XFe96 Bioanalyzer (Agilent Technologies, Santa Clara, CA, USA). After assays, cells were stained with 1:2000 diluted Hoechst dye (Thermo Fisher Scientific, Waltham, MA, USA) using Cytation 5 plate reader (BioTek Instruments) to normalize the readings for the cell count in each well. Measurements were analyzed using XFe96 analyzer's Wave 2.4.0 software (Agilent Technologies), and for MitoStress included calculation of mitochondrial basal respiration, maximal respiration, and adenosine triphosphate (ATP) production. The FuelFlex test included measurements of glucose, fatty acid and glutamine usage as fuels. Each experiment was repeated three times on 20 to 30 technical replicas (wells per group).

### Statistical analysis

Data are presented as the means ± SD. Statistical analysis was performed using either two‐tailed unpaired Student's *t* test to compare two groups or one‐way ANOVA followed by Tukey's post hoc test for comparison of more than two groups. Analyses were performed with assistance of GraphPad Prism 8.3 (GraphPad, La Jolla, CA, USA) software package. Statistical differences with *p* < 0.05 were considered significant. The significant *p* values are provided for each graph above horizontal lines indicating compared groups.

## Results

### Obesity precedes development of low bone mass in CPE KO mice

**Fig 1:**
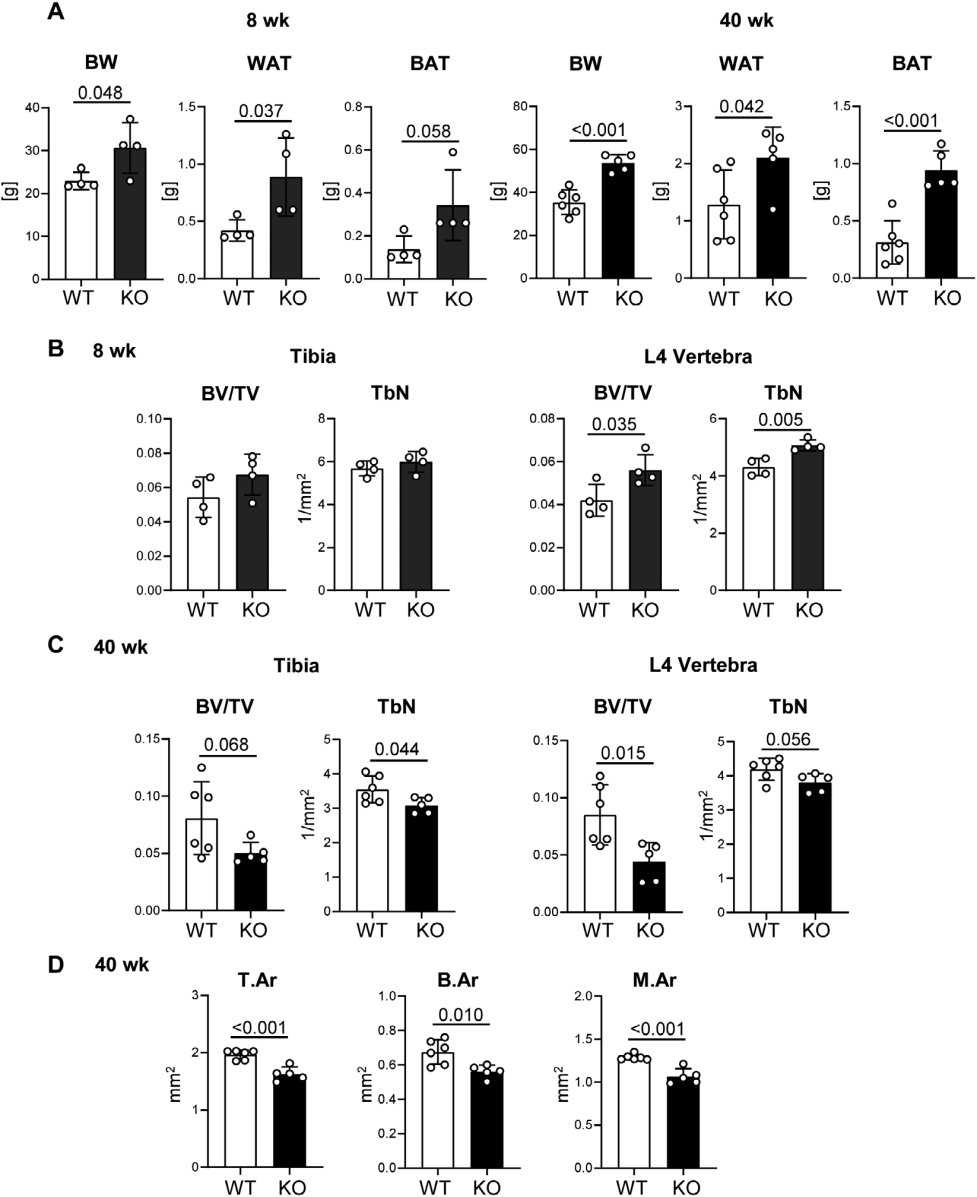
Carboxypeptidase E (CPE) KO male mice develop obesity at early age; they develop a trabecular bone deficit at older age. (*A*) Body weight and weights of epididymal white adipose tissue (WAT) and interscapular brown adipose tissue (BAT) in young (8 weeks) and old (40 weeks) WT and CPE KO mice. (*B*) μCT analysis of trabecular bone in proximal tibia and L4 vertebrae of young mice. (*C*) μCT analysis of trabecular bone in proximal tibia and L4 vertebrae of old mice. (*D*) μCT analysis of tibia cortical bone of old mice. BV/TV = bone volume per tissue volume; TbN = trabecular number; T.Ar = cortical bone total area; B.Ar = cortical bone area; M.Ar = marrow area in diaphysis; young: WT and CPE KO *n* = 4 mice per group; old: WT *n* = 6, CPE KO *n* = 5 mice per group.

It has been reported previously that 40‐week‐old CPE KO mice have low bone mass, in addition to metabolic impairments characterized by obesity, insulin resistance, and infertility.^(^ ^7^ ^)^ To determine whether metabolic and skeletal phenotype of these mice develops simultaneously, we analyzed 8‐week‐old and 40‐week‐old male mice. At 8 weeks of age, CPE‐deficient animals are heavier and have more fat as compared with age‐matched controls (Fig. 1*A*). The obesity phenotype of CPE KO mice becomes more prominent in 40‐week‐old animals (Fig. 1*A*).

Eight‐week‐old CPE KO mice have either normal (tibias) or higher (vertebrae) trabecular bone mass as compared with control WT animals (Fig. 1*B*). This is in a sharp contrast to skeletal phenotype of 40‐week‐old mice, which have significantly lower trabecular bone mass in both tibias and vertebrae (Fig. 1*C*). Older CPE KO mice also have smaller bone as compared with age‐matched WT control mice. Total area, bone area, and marrow area are significantly lower in tibia diaphysis of CPE KO mice (Fig. 1*D*). Thus, CPE deficiency leads to development of obesity at an early age, which is not paralleled with development of low bone mass. The low bone mass develops in older age either as a consequence of an accrual of pathologic effects of dysregulated energy metabolism or independently of CPE enzymatic activities regulating energy metabolism.

### Bone mass is under control of CPE nonenzymatic activities, whereas bone marrow adipocyte tissue is under control of CPE enzymatic activities

**Fig 2:**
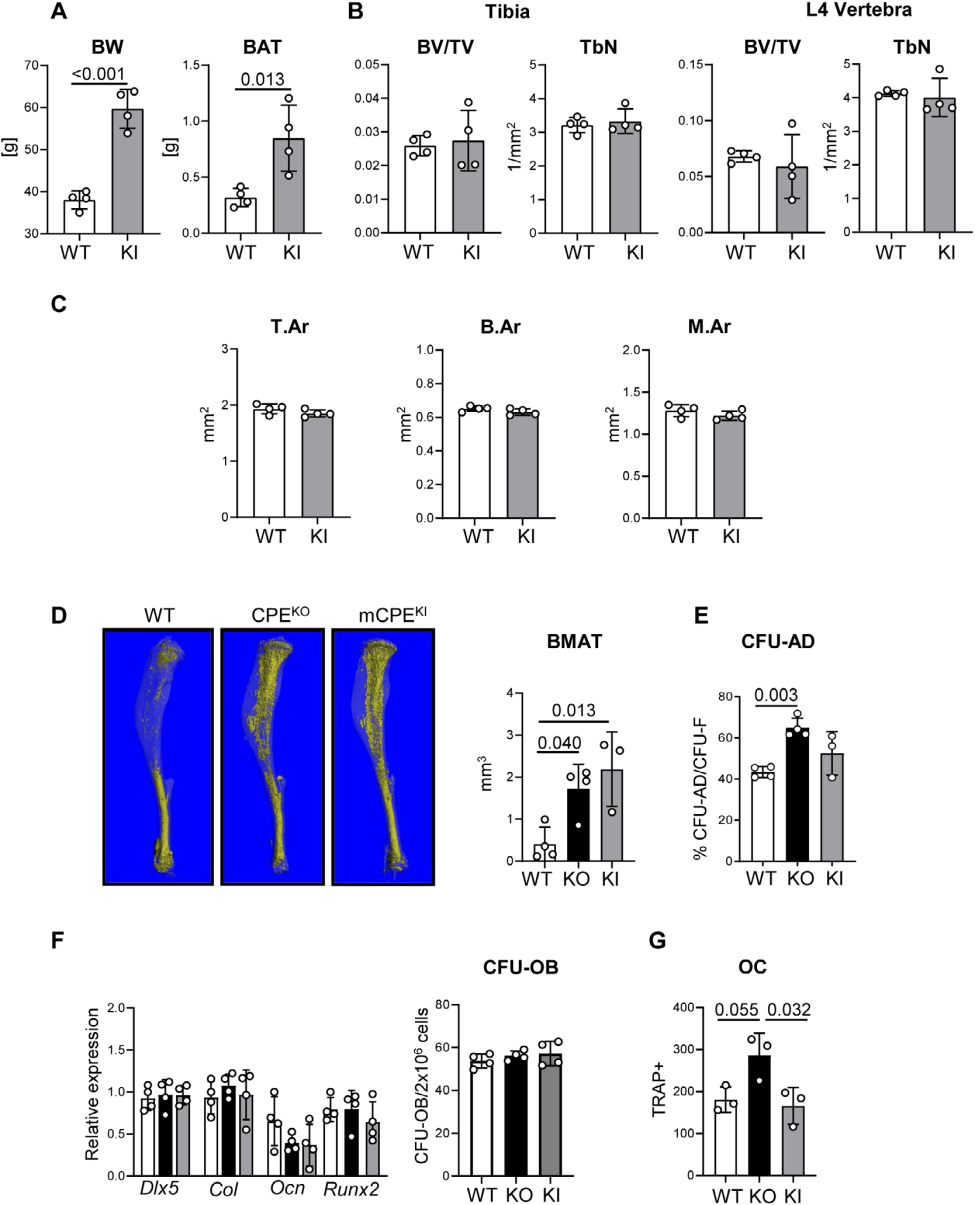
Mutated carboxypeptidase E knockin (mCPE KI) male mice are obese, but do not display trabecular bone deficit at the age of 40 weeks. (*A*) Body weight and weights of brown adipose tissue in WT and mCPE KI males. (*B*) μCT analysis of trabecular bone in proximal tibia and L4 vertebrae. BV/TV = bone volume per tissue volume; TbN = trabecular number. (*C*) μCT analysis of tibia cortical bone. T.Ar = cortical bone total area; B.Ar = cortical bone area; M.Ar = marrow area in diaphysis. (*D*) μCT renderings of BMAT stained with osmium tetroxide in a whole tibia and volumetric measurements of bone marrow adipose tissue (BMAT) in proximal tibia of WT, CPE KO, and mCPE KI mice. (*E*) Bone marrow stromal cell differentiation to adipocytes measured in colony‐forming units for adipocytes (CFU‐AD) assay. (*F*) Osteoblast‐specific gene markers expression in bone marrow stromal cell and osteoblastic differentiation measured in colony‐forming units for osteoblasts (CFU‐OB) assay. (*G*) Marrow nonadherent cell differentiation to tartrate‐resistant acid phosphatase‐positive (TRAP+) multinucleated osteoclast‐like cells (OC) in the presence of 50 ng/mL RANKL and 50 ng/mL macrophage colony‐stimulating factor. White bars = WT; black bars = CPE KO; gray bars = CPE KI; *n* = 4 mice per group.

To determine a contribution of CPE enzymatic and nonenzymatic activities to the regulation of bone mass, we analyzed mCPE KI mice that ectopically expressed CPE‐E342Q protein mutated in the enzymatic domain on the background of CPE KO mice. Consistent with a lack of enzymatic activity, mCPE KI mice are metabolic phenocopies of CPE KO mice and are characterized by high body weight and increased fat content (Fig. 2*A*). However, in contrast to CPE KO mice, 40‐week‐old mCPE KI mice have trabecular bone mass and trabeculae number in tibia and in vertebrae similar to WT animals at the same age (Fig. 2*B*). Similarly, their cortical bone mass is rescued and not different from WT animals (Fig. 2*C*). This suggests that the CPE nonenzymatic activity is sufficient for the maintenance of trabecular and cortical bone mass. In contrast, volumes of bone marrow adipose tissue (BMAT) in tibia of mCPE KI and CPE KO mice were similar and much higher as compared with the volumes of BMAT accumulated in WT mice (Fig. 2*D*). Moreover, primary BMSCs’ potential to form colonies of fat‐laden cells (CFU‐AD) was also increased (Fig. 2*E*). This implies that BMAT accumulation is under control of CPE enzymatic activities.

Next, we compared the potential of mesenchymal and hematopoietic progenitors derived from WT, CPE KO, and mCPE KI to differentiate toward osteoblasts and osteoclasts, respectively. As shown in Fig. 2*F*, differentiation of mesenchymal BMSC toward osteoblasts was not affected by the status of endogenous CPE. Neither the osteoblast gene markers’ expression nor a number of mineralized CFU‐OBs developed from BMSCs of WT, CPE KO, or mCPE KI mice were different (Fig. 2*F*). However, in the presence of RANKL and M‐CSF, the nonadherent marrow cells derived from CPE KO mice differentiated abundantly toward multinucleated TRAP+ osteoclast‐like cells indicating large number of osteoclast progenitors (Fig. 2*G*). In contrast, osteoclast differentiation from nonadherent cells derived from bone marrow of mCPE KI mice was at the level of WT differentiation. These data suggest that CPE expressed in cells of osteoclast lineage negatively regulates osteoclast differentiation and that this effect is independent of CPE enzymatic activity.

### CPE protein regulates cellular energy metabolism in cells of osteoblastic lineage

**Fig 3:**
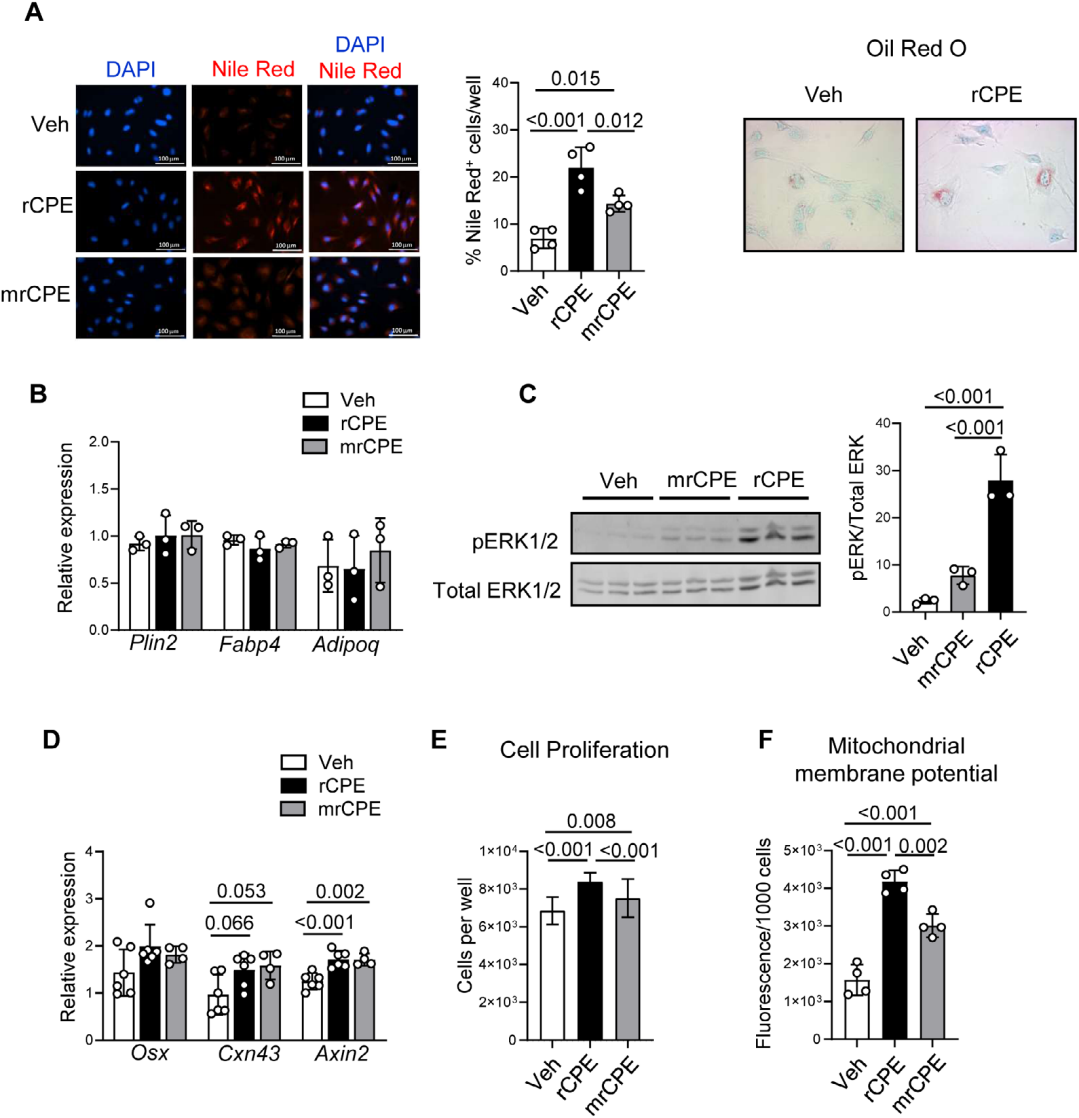
Effect of treatment with either of nonmutated recombinant carboxypeptidase E (rCPE) or mutated recombinant CPE (mrCPE) on preosteoblastic U33 cells. (*A*) Perinuclear lipid accumulation after 24‐hour treatment detected with either fluorescent Nile red or oil red O staining, as indicated. Fractions of cells positive for Nile red staining were quantified and presented on the graph. (*B*) Relative mRNA expression of adipocyte‐specific gene markers after 72‐hour treatment (*n* = 4 to 6 independent RNA samples per treatment). (*C*) Phosphorylation of ERK1/2 kinases after 10‐min treatment. Thirty μg of protein lysate was loaded per lane. pERK1/2 and total ERK1/2 were detected as described in the Materials and Methods section. Relative band densities were measured with Image J and measurements representing pERK1/2 were normalized to the density of corresponding total ERK1/2. (*D*) Relative mRNA expression of osteoblast‐specific gene markers after 72‐hour treatment (*n* = 4 to 6 independent RNA samples per treatment). (*E*) The effect of 24‐hour treatment on cell proliferation measured by Cytation 5 (*n* = 9 to 17 replicas per treatment). (*F*) The effect of 24‐hour treatment on mitochondrial activity measured as mitochondrial membrane polarization using tetramethylrhodamine ethyl ester perchlorate (TMRE) assay (*n* = 4 replicas per treatment). White bars = vehicle (Veh); black bars = rCPE; gray bars = mrCPE.

There is increasing evidence that in neuronal and cancer cells CPE may act as an endocrine/paracrine signaling factor, independent of its enzymatic and endogenous activities. Therefore, we have tested whether SSCs respond to treatment with exogenous CPE protein. In these experiments, we used two preosteoblastic cell lines, U33 and MC3T3 (not shown), which were treated with either intact (rCPE) or mutated (mrCPE) recombinant CPE. We found that cells exposed for 24 hours to either rCPE or mrCPE accumulated lipid droplets in the perinuclear cell compartment (Fig. 3*A*). Accumulation of lipids was transient and not associated with increased expression of adipocyte‐specific gene markers (Fig. 3*B*). Consistent with osteoblastic phenotype, U33 and MC3T3 cells did not express perilipin 1, which codes for a protein necessary for the formation of adipocytic lipid vacuoles; however, similar to other osteoblastic cells, they did express perilipin 2 (*Plin2*).^(^ ^23^ ^)^ Accumulation of lipid droplets was not associated with increased expression of *Plin2*, or of *Fabp4* and *Adipoq*, two bona fide adipocyte gene markers (Fig. 3*B*), as well as metabolic markers such as *Ucp1*, *Dio2*, and *Cpt1a* (Supplementary Fig. S1*A*).

Treatment with CPE proteins increased phosphorylation of ERK1/2 signaling kinases with a role in regulation of osteoblastic differentiation and cell metabolism (Fig. 3*C*). Both rCPE and mrCPE proteins induced pERK1/2; however, treatment with mrCPE required a longer time to achieve, similar to rCPE effect on the level of pERK1/2 (Supplementary Fig. S2). The transient lipid accumulation and activation of ERKs correlated with modestly increased expression of two markers of the Wnt‐signaling pathway, *Cxn43* and *Axin2*, and a tendency to increase in expression of osteoblast‐specific transcription factor osterix (*Osx*; Fig. 3*D*). However, no significant differences were noticed at the levels of expression of other osteoblastic markers such as *Dlx5*, *Runx2*, *Col1*, *osteocalcin*, *Bmp4*, and *Wnt10b* (Supplementary Fig. S1*B*). In addition, exogenous CPE increased proliferation of treated cells (Fig. 3*E*). Importantly, treatment with either rCPE or mrCPE was associated with increased mitochondrial membrane potential detected by TMRE accumulation, a measure indicating mitochondrial activity (Fig. 3*F*).

**Fig 4:**
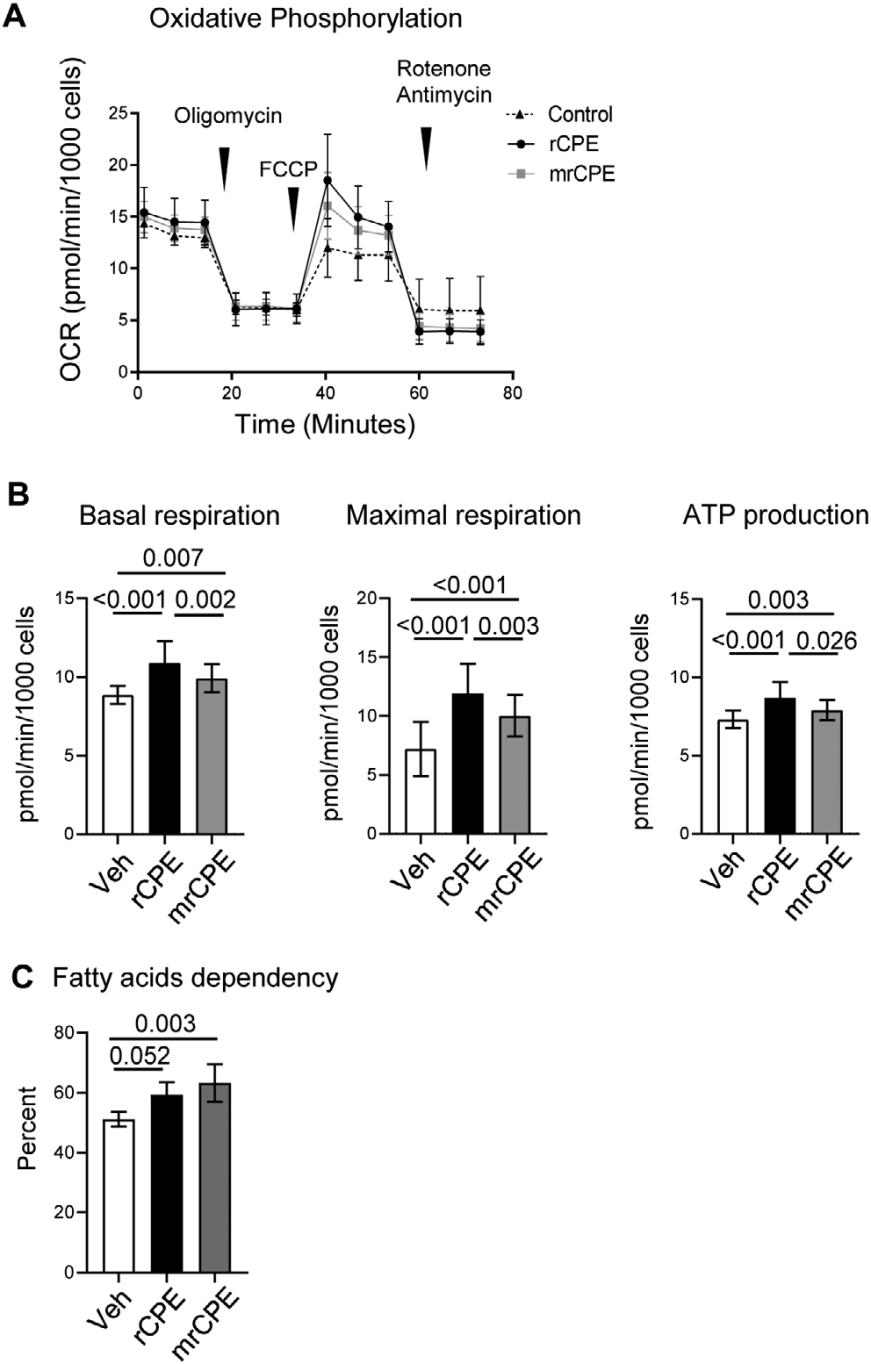
Nonmutated recombinant carboxypeptidase E (rCPE) and mutated recombinant CPE (mrCPE) regulate cellular bioenergetics in U33 cells. (*A*) Profile of oxygen consumption (oxidative phosphorylation) measured with MitoStress assay after 24‐hour treatment with either rCPE or mrCPE. (*B*) Calculated rates of basal respiration, maximal respiration, and adenosine triphosphate production measured with MitoStress assay, as above. (*C*) An effect of rCPE and mrCPE on fatty acids dependency of U33 cells measured with FuelFlex assay. White bars = Vehicle (Veh); black bars = rCPE; gray bars = mrCPE. Both assays were repeated three times with 20 to 30 technical replicas per group; representative results of one assay are shown.

Cellular metabolism and ATP production by mitochondria regulates cell differentiation and function. The effect of both CPE proteins on mitochondrial function was measured using Seahorse XF Mito Stress assay. Consistent with an increase in mitochondrial activity, treatment of U33 cells with either rCPE or mrCPE protein increased oxidative phosphorylation (Fig. 4*A*). This resulted in increased basal and maximal respiration, and ATP production (Fig. 4*B*). To determine whether increased cellular energy metabolism in basal energetic state is associated with changes in oxidization of three major mitochondrial fuels: glucose, fatty acids, or glutamine, the Seahorse FuelFlex assay was performed. Consistently, treatment with either rCPE or mrCPE proteins increased cell dependency to use fatty acids (Fig. 4*C*). The use of two other fuels, glucose and glutamine, was inconsistent in the repeated assays; therefore, these results are not presented.

### *Cpe* expression is under negative control of PPARγ2 adipocyte‐specific transcription factor and is upregulated in primary human SSCs during osteoblastic differentiation

Previous analysis of murine SSC transcriptome^(^ ^24^ ^)^ (the data are deposited in NCBI Gene Expression Omnibus, accession number GSE10192) showed that CPE mRNA expression is under negative control of proadipocytic PPARγ2 transcription factor. Ectopic expression of PPARγ2 in U33 cells downregulated *Cpe* expression by sevenfold, as compared with the same cells transfected with an empty expression vector (*p* = 0.000007). Moreover, activation of PPARγ2 with rosiglitazone downregulated *Cpe* expression by additional 2.7‐fold (*p* = 0.00002; Supplementary Table S2). Based on the inverse relationship between adipocyte and osteoblast differentiation, this observation suggests a positive correlation between CPE and osteoblast differentiation. Indeed, *Cpe* mRNA expression correlates positively with the osteoblastic potential of human SSCs. Human SSCs were grown from bone tissue specimens collected from different skeletal locations during orthopedic procedures. As shown in Table 1 and described in the Materials and Methods section, SSCs were cultivated from bone marrow collected from either a femoral head, which was highly enriched in adipocytes (yellow marrow, YB cell line), or marrow collected from a knee representing hematopoietic marrow (red marrow, MR cell line), or cell outgrowth from knee cartilage (CT cell line), or marrow aspirates collected from iliac crest of two different subjects (MA3 and MA4 cell lines).

**Fig 5:**
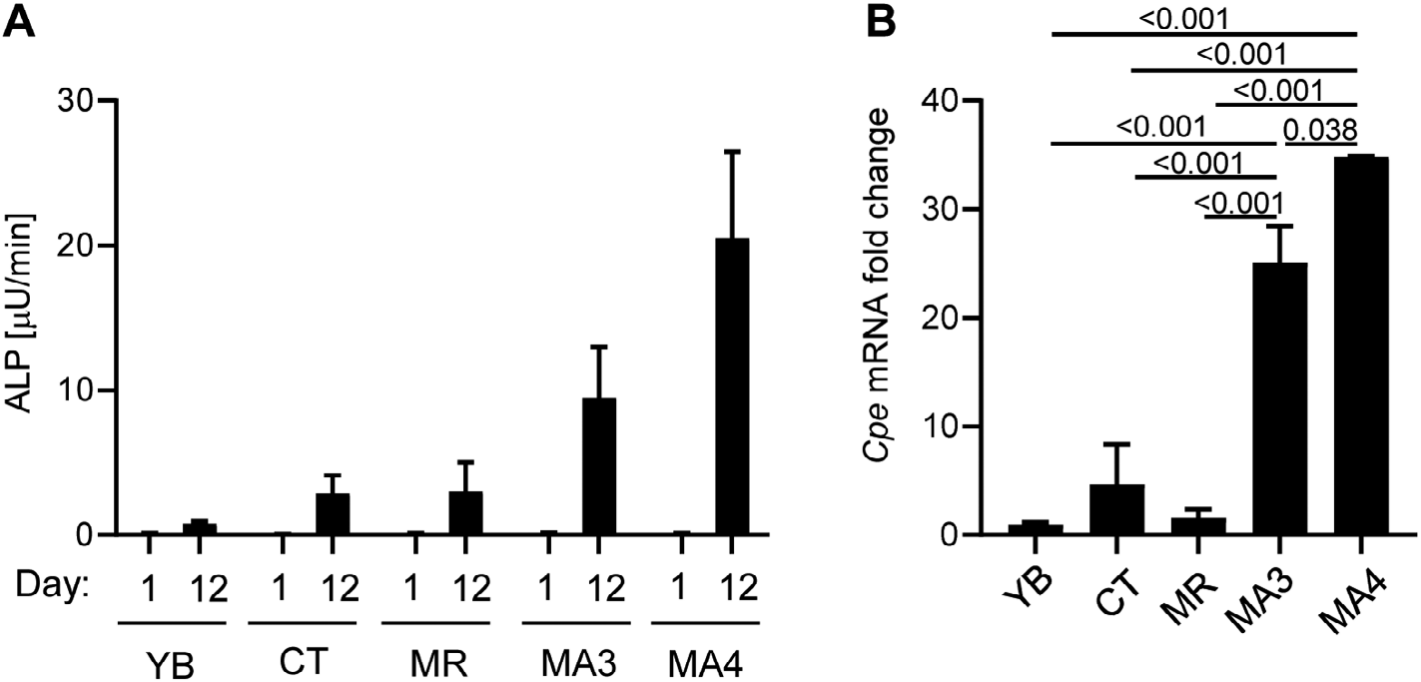
Human skeletal stem cells (SSCs) osteoblastic differentiation correlates with increased expression of carboxypeptidase E (CPE). (*A*) Alkaline phosphatase enzyme activity of human SSCs at day 1 (D1) and after 12 days of growth (D12) in media supplemented with β‐glycerol phosphate (10mM), ascorbic acid (50 μg/mL), and dexamethasone (10nM). (*B*) The same SSCs were grown as in (*A*), and RNA was isolated at D12 and analyzed with RT‐PCR for human *Cpe* mRNA expression. ALP = alkaline phosphatase; CT = cell outgrowth from knee cartilage; MA3 and MA4 = marrow aspirates collected from iliac crest of two different subjects; MR = marrow collected from a knee representing hematopoietic marrow; YB = marrow collected from a femoral head highly enriched in adipocytes.

In basal conditions, ALP enzyme activity, the bona fide marker of osteoblast phenotype, was very low and similar in all five cell lines (Fig. 5*A*). Twelve days of growth in osteogenic media resulted in the highest increase in ALP activity in MA3 and MA4, a modest increase in CT and MR, and minimal change in the YB cell line (Fig. 5*A*). The increase in ALP activity correlated positively with *Cpe* expression, which increased highly in MA3 and MA4 cells (28‐fold in MA3 and 35‐fold in MA4) and either not changed (YB) or moderately increased in CT (3.9‐fold) and MR (1.5‐fold) cells (Fig. 5*B*). The increase in MA3 and MA4 cells of both ALP activity and *Cpe* expression correlated with an increase in mRNA levels of other genes associated with osteoblast differentiation and bone formation, including decorin (*Dcn*), lysyl oxidase like 4 (*Loxl4*), collagen 12A1 (*Col12A1*), lumican (*Lum*), and collagen 1 (*Col1A*; Table 2).

**Table 2:** Fold‐Change in Expression of Gene Biomarkers in Human Skeletal Stem Cell Isolates After 12 Days of Growth in Osteogenic Conditions

Gene markers	Bone marrow isolates				
	YB	CT	MR	MA3	MA4
*Cpe*	−1.02	3.95	1.47	28.0	34.81
*Dcn*	2.48	2.20	7.29	20.76	26.52
*Loxl4*	1.82	20.08	−3.63	8.19	14.07
*Col12A1*	−6.29	2.74	5.88	24.03	9.32
*Lum*	−2.21	1.31	1.76	8.45	4.71
*Col1A*	−1.23	−52.93	−1.55	4.31	2.60

CT = cell outgrowth from knee cartilage; MA3 and MA4 = marrow aspirates collected from iliac crest of two different subjects; MR = marrow collected from a knee representing hematopoietic marrow; YB = marrow collected from a femoral head highly enriched in adipocytes.

## Discussion

These studies present an analysis of CPE multiple functions, supporting differentiation and metabolism of bone cells that define bone remodeling and bone mass. We have described the role of enzymatic, nonenzymatic, and trophic activities of CPE in the regulation of bone homeostasis. These activities are summarized in Table 3. First, we have shown that the same peptidase activity, which produces mature hormones and neuropeptides and regulates systemic energy metabolism and function of adipose tissue, also regulates expansion of bone marrow adipose tissue independently from mechanisms regulating bone mass. Thus, CPE lacking enzymatic activity renders animals obese and increases BMAT volume in long bones without affecting bone mass. Second, the nonenzymatic activity of endogenous CPE in marrow cells of hematopoietic lineage negatively regulates osteoclast development. Thus, increased osteoclast differentiation in ex vivo cultures of nonadherent cells isolated from the marrow of CPE KO mice is completely reverted in the cells isolated from the marrow of mCPE KI mice carrying mutation in the enzymatic domain. Third, in cells of osteoblastic lineage, CPE trophic nonenzymatic activity induces ERK1/2 phosphorylation, mitochondrial activity, and ATP production, and increases cellular dependence on fatty acids as fuel for energy production. This, together with a positive association of *Cpe* expression with a potential of human SSCs to acquire osteoblastic phenotype and a negative association with adipogenesis, suggests that at the cellular level CPE protein may contribute to the allocation of SSCs toward osteoblastic lineage. Presented studies are limited to male mice based on difficulties in obtaining a statistically representative group of females because CPE KO and mCPE KI mice are infertile. However, in our previously published study, we showed that both CPE KO males and females are obese and have low bone mass.^(^ ^7^ ^)^ The most recent studies have shown that both sexes of mCPE KI mice have the same neurological phenotype (YPL, personal information), increasing the probability that CPE skeletal activities are similar in males and females.

**Table 3:** Summary of Carboxypeptidase E (CPE) Activities Regulating Bone Mass and Energy Balance

CPE activities	Metabolic and skeletal functions
Enzymatic carboxypeptidase activity	Energy balance, negative regulation of obesity and bone marrow adipocyte tissue accumulation
Endogenous nonenzymatic activity	Negative regulation of osteoclast differentiation
Trophic nonenzymatic activity	Regulation of osteoblast bioenergetics and ERK1/2 signaling

Our study uncovered unknown skeletal functions of CPE, placing this protein among other common regulators of bone and energy metabolism. Low bone mass of animals deficient in CPE protein had been previously attributed to alterations in efferent hypothalamic and hormonal signaling to bone‐regulating bone resorption.^(^ ^7^ ^)^ The low bone mass of CPE KO mice was credited to the lack of enzymatic carboxypeptidase activity leading to the alterations in the complex interplay between peptides produced in the hypothalamus (CART, NPY, αMSH) that are involved in regulation of energy metabolism and bone remodeling.^(^ ^7^ ^)^ However, our study—showing that nonenzymatic activity of CPE directly regulates osteoclast differentiation—suggests that, at least in part, CPE deficiency in osteoclasts contributes to the low bone mass of CPE KO mice. Importantly, osteoclast differentiation was normal in hematopoietic cells that expressed a mutated form of CPE pointing to the nonenzymatic activity negatively regulating osteoclast differentiation.

These results are in contrast to the findings by Kim and colleagues, who suggested the opposite effect, namely that CPE is enhancing osteoclast formation.^(^ ^14^ ^)^ It has been shown that CPE protein levels increase in the isolated bone marrow macrophages (BMMs) during their osteoclast differentiation in the ex vivo cultures. In addition, retroviral overexpression of CPE in BMMs increased formation of multinucleated TRAP+ osteoclasts in the presence of M‐CSF and RANKL, and enhanced the induction of c‐Fos and NFATc1, osteoclast‐specific transcription factors. The different conditions and gene dosage may account for a discrepancy between results of their study and our study. In our model, instead of using purified BMMs and high levels of CPE delivered by retroviral expression, we used a fraction of bone marrow nonadherent cells, which either did not express CPE or expressed mutated CPE at the physiological levels. Thus, the source of osteoclast progenitors and CPE levels may explain the differences between these studies. Nevertheless, these data suggest that CPE acts as a gatekeeper for osteoclast differentiation, and that peptidase activity of CPE is not involved in this process. Of note, a new human study, which correlated genome‐wide signal for estimated BMD with osteoclast expression quantitative trait loci, identified *CPE* as a gene associated with osteoporosis.^(^ ^25^ ^)^

Finding that CPE enzymatic activity controls BMAT accumulation in long bones has several implications. BMAT accumulation may be under the same central control regulating metabolism in peripheral adipose tissues. Both CPE^*fat/fat*^ and CPE KO mice are morbidly obese, have decreased basal metabolic rate, reduced utilization of lipids for energy production, reduced spontaneous activity, and are hyperphagic. They have low levels of anorexigenic αMSH caused by deficient POMC processing.^(^ ^15^ ^)^ Thus, it is possible that systemic changes in energy metabolism, which increase fat accumulation in peripheral fat depots, are affecting bone marrow adipocytes by the same mechanism. On the other hand, a number of studies suggest that marrow adipocyte development is under the control of the sympathetic nervous system acting directly on marrow cells. BMAT is innervated by sympathetic neurons and shares the same pathways with the hypothalamus and with peripheral adipose tissue.^(^ ^26^, ^27^ ^)^ Moreover, it is also possible that αMSH directly regulates marrow adipocyte. The circulating αMSH increases lipolysis by acting on melanocortin 5 receptors present in adipocytes,^(^ ^28^ ^)^ including marrow adipocytes (BLC, personal information). Thus, a decrease in αMSH signaling may impair lipolysis and may favor lipid accumulation in marrow adipocytes. Another possibility is that CPE expressed in SSCs plays a role in its lineage allocation toward adipocytes, independently of its systemic effects, as we have shown that the adipocyte‐specific transcription factor PPARγ2 negatively regulates *Cpe* expression, and that SSC potential to form fat‐laden colonies is increased in BMSCs lacking CPE enzymatic activity. Taken together, BMAT is under negative control of CPE enzymatic activity, which may comprise an involvement of central control, SNS signaling, and CPE activity directly in SSCs. Of note is the high adipocyte accumulation with a lack of an effect on bone mass observed in mCPE KI, which is somewhat surprising. However, it provides additional evidence that a reciprocal relationship between adipocyte and osteoblast differentiation may be an exception, not a rule, and supports the existence of regulatory mechanisms specific for marrow adipocytes, which are separate from mechanisms regulating osteoblast differentiation.^(^ ^29^ ^)^

Besides systemic energy metabolism, CPE also regulates energy metabolism in cells of osteoblast lineage, which uncovers another novel function of this “old” protein. The nonenzymatic, trophic activity of CPE has been already shown to regulate neuronal stem cells differentiation and survival, and involves ERK activation and WNT signaling.^(^ ^11^ ^)^ Here, we have shown for the first time that in cells of mesenchymal lineage, extracellular CPE activates ERKs, increases cell proliferation, and increases mitochondrial activity, resulting in increased ATP production and increased cell dependency on fatty acids as an energy source. In addition to the analysis of bioenergetics in the murine system, we have positively correlated increased expression of *Cpe* mRNA with human SSC osteoblast‐like phenotype and expression of transcripts coding for proteins involved in the biogenesis of connective tissues and components of bone matrix. Based on these findings and CPE's role in the regulation of cellular bioenergetics and fuel choice, we hypothesize that CPE participates in processes regulating SSC commitment to osteoblasts.

There is an additional aspect of CPE biology that needs to be discussed. Based on our results and others’ reports, there is an indication that CPE activities regulating signaling and bioenergetics in mesenchymal versus neuronal versus cancer cells may have opposite effects. For example, in cancer cells, CPE increases oxidative phosphorylation, but in contrast to our findings, it enhances glucose flux, not fatty acids, in the tricarboxylic acid cycle.^*(*^ ^30^ ^)^ Another example includes the regulation of *Cpe* expression by PPARγ in neurons and mesenchymal cells. Peroxisome proliferator response element (PPRE) sequences have been found in the *Cpe* promoter region of rats, mice, and humans; and it has been shown that these sequences positively regulate promoter activity in neuronal cells.^(^ ^10^ ^)^ In support of this regulation, we have shown previously that PPARγ, upon activation with full agonist rosiglitazone upregulates *Cpe* expression in rat hippocampal neurons, which had antidepressive effects on rats.^(^ ^6^ ^)^ However, here we showed that in cells of mesenchymal lineage, PPARγ downregulates *Cpe* expression, which is consistent with in vitro and in vivo evidence linking CPE to the negative regulation of adipogenesis. Thus, multifunctional characteristics of CPE protein are determined by cell type, which emphasizes a unique spectrum of CPE activities in the skeleton.

In conclusion, CPE integrates bone metabolism with energy metabolism at the level of enzyme‐dependent and enzyme‐independent activities. CPE regulates energy metabolism and bone mass on multiple cellular levels and by different mechanisms providing additional evidence for close ties between bone metabolism and energy metabolism.

## Disclosures

All authors state that they have no conflicts of interest.

## Author contributions

**Amit Chougule:** Conceptualization; data curation; formal analysis. **Vipula Kolli:** Conceptualization; data curation; formal analysis. **Sudipta Baroi:** Conceptualization; data curation; formal analysis. **Nabil Ebraheim:** Data curation; formal analysis. **Piotr Czernik:** Data curation; formal analysis. **Y. Peng Loh:** Conceptualization; data curation; formal analysis; supervision; writing‐review and editing. **Beata Lecka‐Czernik:** Conceptualization; data curation; formal analysis; supervision; writing‐original draft; writing‐review and editing.

### Peer Review

The peer review history for this article is available at https://publons.com/publon/10.1002/jbm4.10392.

## Supporting information


**Appendix S1**: Supporting information.
**Supplementary Fig. S1**. Effect of rCPE and mrCPE treatment of U33 cells on expression of of adipocyte (A) and osteoblast (B) gene markers.
**Supplementary Fig. S2**. Western blot analysis of pERK1/2 and ERK1/2 levels after treatment with rCPE and mrCPE for different time. Image J quantification of bands density
**Supplementary Fig. S3**. Full western blot images of pERK1/2 and ERK1/2 after treatment with rCPE and mrCPE for 10 min (for Fig. 3C).
**Supplementary Table S1**. List of primers used for gene expression analysis in murine and human cells
**Supplementary Table S2**. PPARg2 downregulates Cpe mRNA expression in murine pre‐osteoblastic cells
