# Supplementary material for: Nonenzymatic and Trophic Activities of Carboxypeptidase E Regulate Bone Mass and Bioenergetics of Skeletal Stem Cells in Mice
Source: JBMR Plus. 2020 Aug 12;4(9):e10392. doi: 10.1002/jbm4.10392 (PMC7507073; doi:10.1002/jbm4.10392)
Supplement: Supplementary file 1 — Appendix S1: Supporting information. Supplementary Fig. S1. Effect of rCPE and mrCPE treatment of U33 cells on expression of of adipocyte (A) and osteoblast (B) gene markers. Supplementary Fig. S2. Western blot analysis of pERK1/2 and ERK1/2 levels after treatment with rCPE and mrCPE for different time. Image J quantification of bands density Supplementary Fig. S3. Full western blot images of pERK1/2 and ERK1/2 after treatment with rCPE and mrCPE for 10 min (for Fig. 3C). Supplementary Table S1. List of primers used for gene expression analysis in murine and human cells Supplementary Table S2. PPARg2 downregulates Cpe mRNA expression in murine pre‐osteoblastic cells [file JBM4-4-e10392-s001.pptx]

## Slide 1
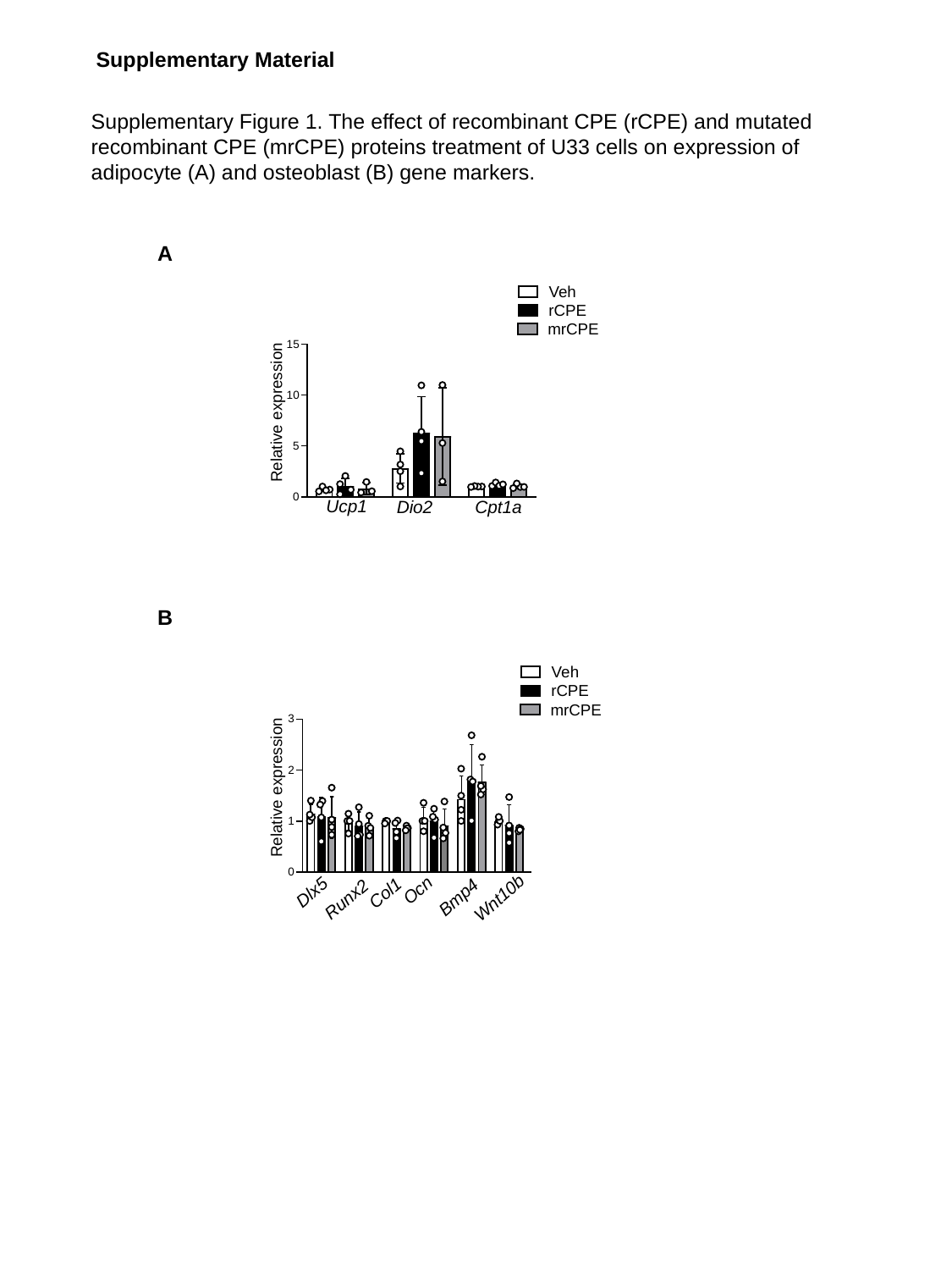

Supplementary Material
Supplementary Figure 1. The effect of recombinant CPE (rCPE) and mutated recombinant CPE (mrCPE) proteins treatment of U33 cells on expression of adipocyte (A) and osteoblast (B) gene markers.
A
Veh
rCPE
mrCPE
Relative expression
Ucp1
Dio2
Cpt1a
B
Veh
rCPE
mrCPE
Relative expression
Dlx5
Col1
Runx2
Ocn
Bmp4
Wnt10b

## Slide 2
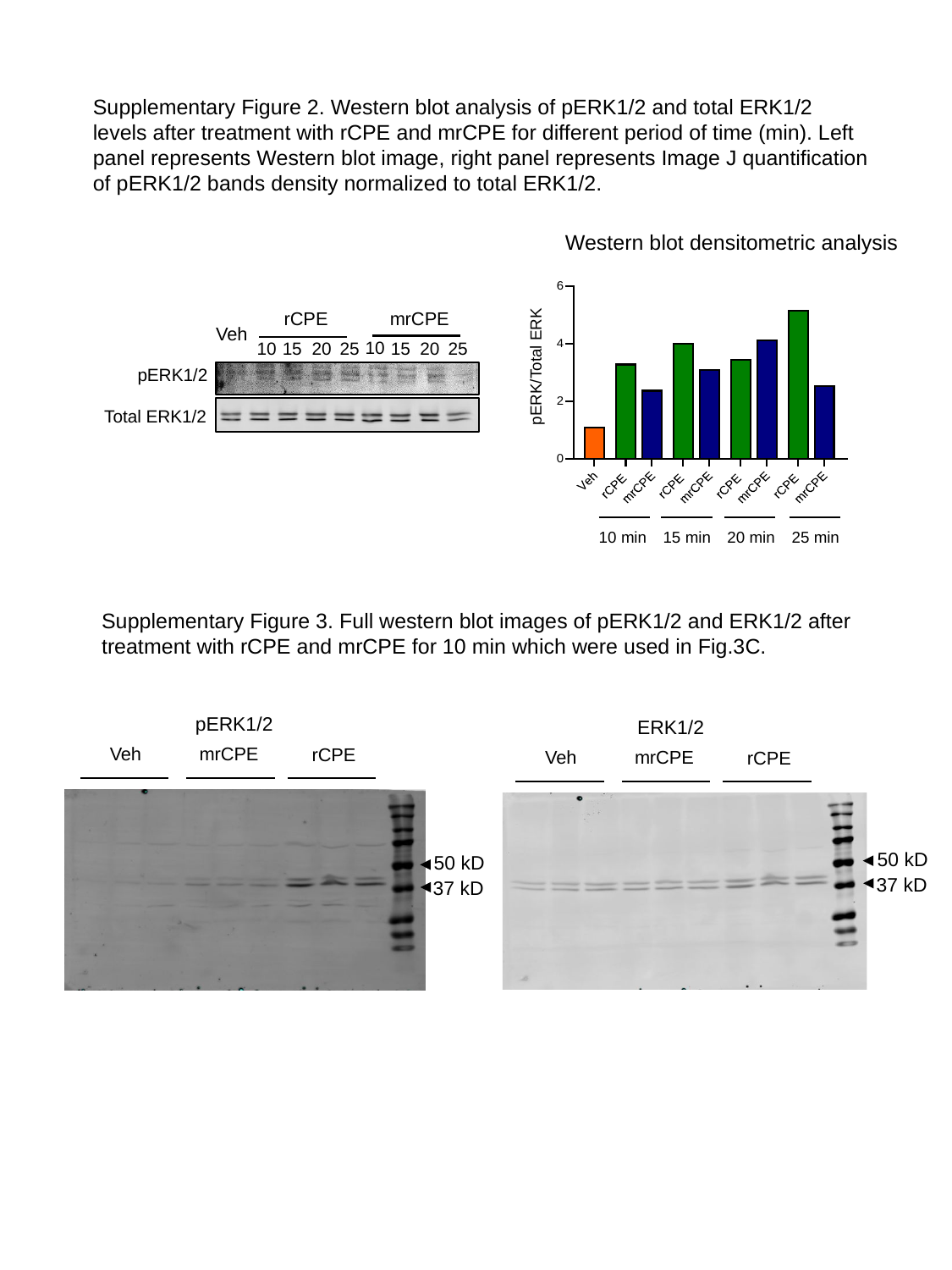

Supplementary Figure 2. Western blot analysis of pERK1/2 and total ERK1/2 levels after treatment with rCPE and mrCPE for different period of time (min). Left panel represents Western blot image, right panel represents Image J quantification of pERK1/2 bands density normalized to total ERK1/2.
Western blot densitometric analysis
pERK/Total ERK
10 min
15 min
20 min
25 min
rCPE
mrCPE
Veh
10
10
15
20
15
25
20
25
pERK1/2
Total ERK1/2
Supplementary Figure 3. Full western blot images of pERK1/2 and ERK1/2 after treatment with rCPE and mrCPE for 10 min which were used in Fig.3C.
pERK1/2
Veh
mrCPE
rCPE
50 kD
37 kD
ERK1/2
Veh
mrCPE
rCPE
50 kD
37 kD

## Slide 3
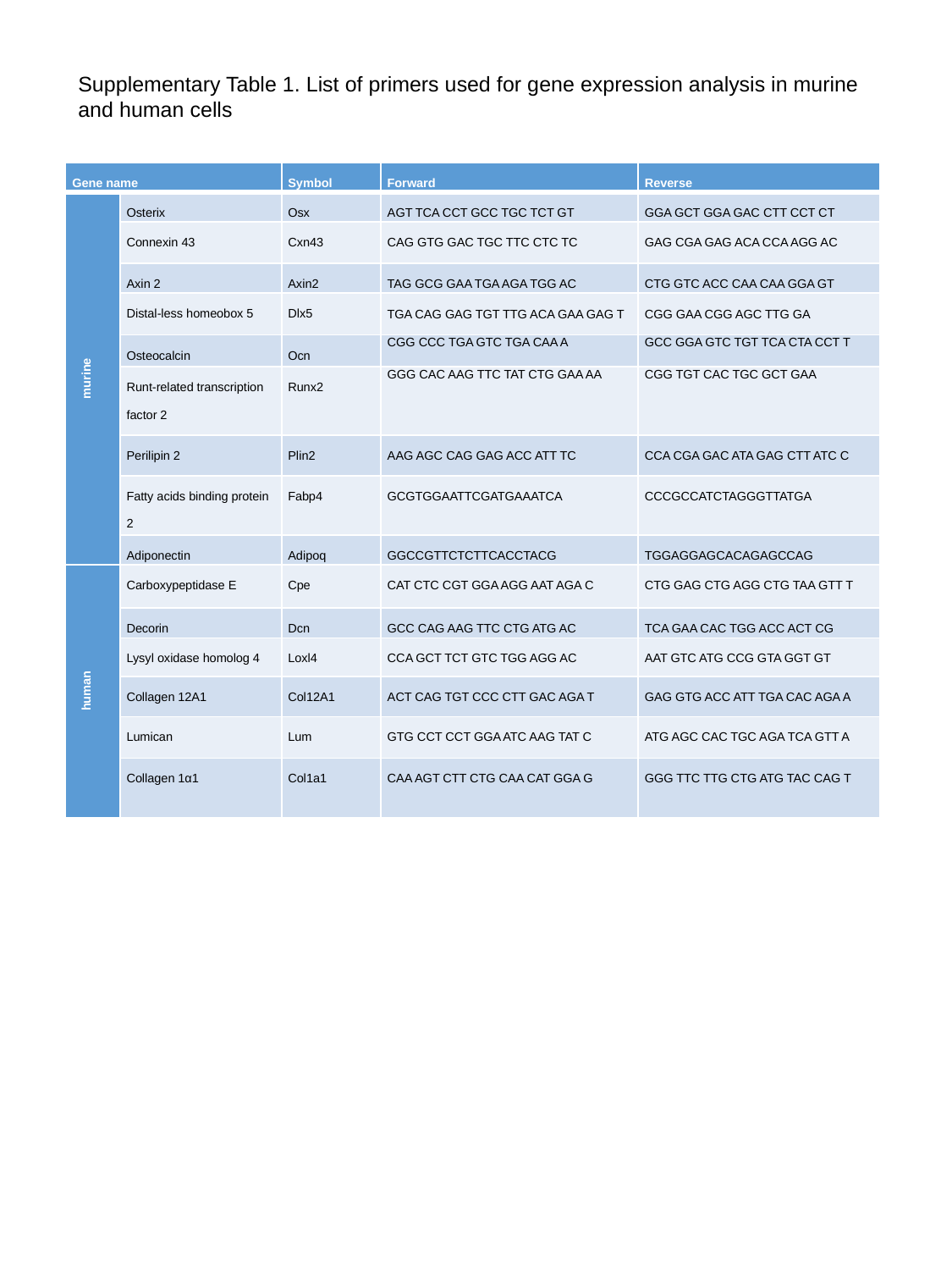

Supplementary Table 1. List of primers used for gene expression analysis in murine and human cells
| Gene name | | Symbol | Forward | Reverse |
| --- | --- | --- | --- | --- |
| murine | Osterix | Osx | AGT TCA CCT GCC TGC TCT GT | GGA GCT GGA GAC CTT CCT CT |
| | Connexin 43 | Cxn43 | CAG GTG GAC TGC TTC CTC TC | GAG CGA GAG ACA CCA AGG AC |
| | Axin 2 | Axin2 | TAG GCG GAA TGA AGA TGG AC | CTG GTC ACC CAA CAA GGA GT |
| | Distal-less homeobox 5 | Dlx5 | TGA CAG GAG TGT TTG ACA GAA GAG T | CGG GAA CGG AGC TTG GA |
| | Osteocalcin | Ocn | CGG CCC TGA GTC TGA CAA A | GCC GGA GTC TGT TCA CTA CCT T |
| | Runt-related transcription factor 2 | Runx2 | GGG CAC AAG TTC TAT CTG GAA AA | CGG TGT CAC TGC GCT GAA |
| | Perilipin 2 | Plin2 | AAG AGC CAG GAG ACC ATT TC | CCA CGA GAC ATA GAG CTT ATC C |
| | Fatty acids binding protein 2 | Fabp4 | GCGTGGAATTCGATGAAATCA | CCCGCCATCTAGGGTTATGA |
| | Adiponectin | Adipoq | GGCCGTTCTCTTCACCTACG | TGGAGGAGCACAGAGCCAG |
| human | Carboxypeptidase E | Cpe | CAT CTC CGT GGA AGG AAT AGA C | CTG GAG CTG AGG CTG TAA GTT T |
| | Decorin | Dcn | GCC CAG AAG TTC CTG ATG AC | TCA GAA CAC TGG ACC ACT CG |
| | Lysyl oxidase homolog 4 | Loxl4 | CCA GCT TCT GTC TGG AGG AC | AAT GTC ATG CCG GTA GGT GT |
| | Collagen 12A1 | Col12A1 | ACT CAG TGT CCC CTT GAC AGA T | GAG GTG ACC ATT TGA CAC AGA A |
| | Lumican | Lum | GTG CCT CCT GGA ATC AAG TAT C | ATG AGC CAC TGC AGA TCA GTT A |
| | Collagen 1α1 | Col1a1 | CAA AGT CTT CTG CAA CAT GGA G | GGG TTC TTG CTG ATG TAC CAG T |

## Slide 4
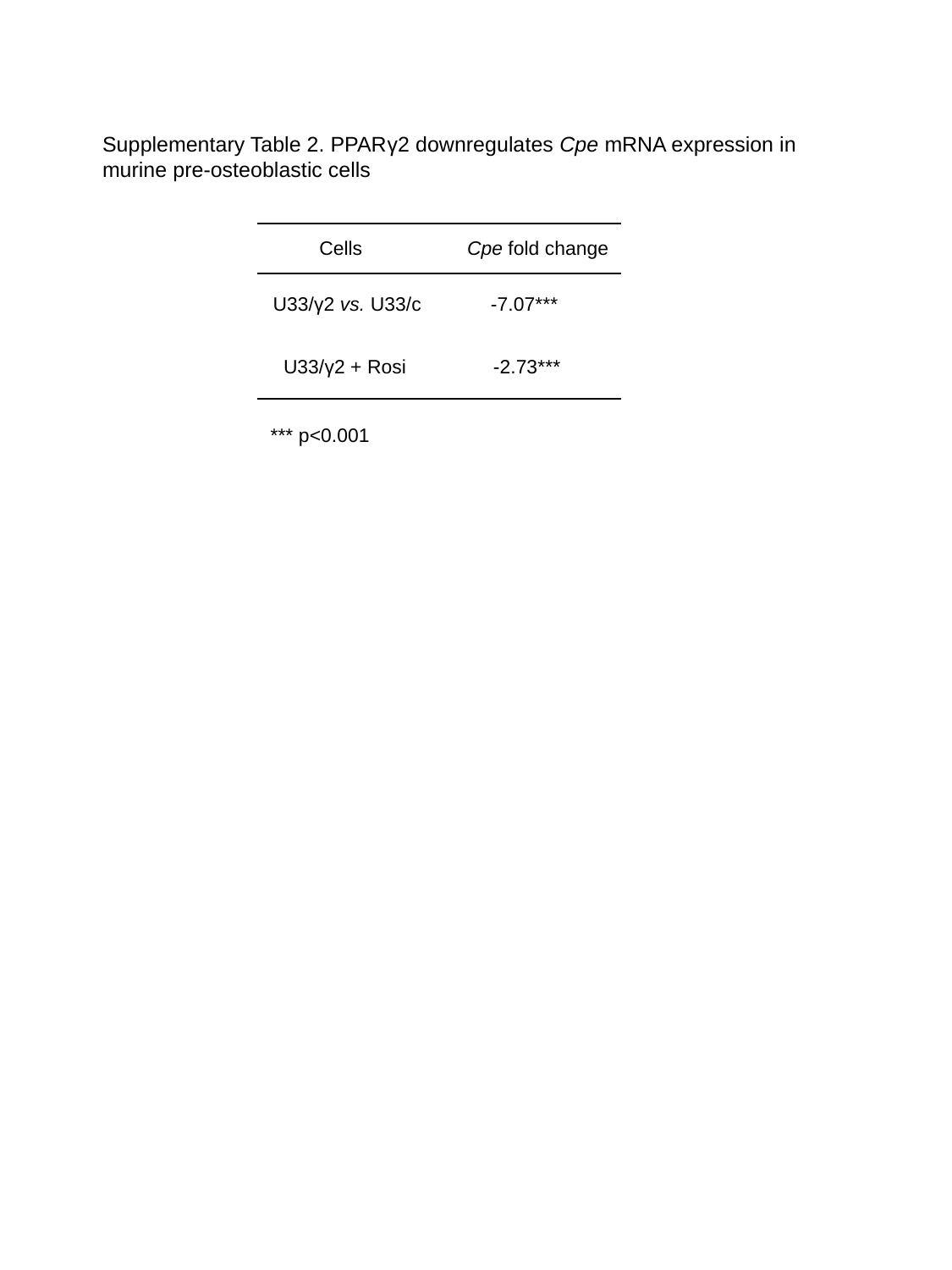

Supplementary Table 2. PPARγ2 downregulates Cpe mRNA expression in murine pre-osteoblastic cells
Cells
Cpe fold change
-7.07***
U33/γ2 vs. U33/c
U33/γ2 + Rosi
-2.73***
*** p<0.001
